# Supplementary material for: A chemical biology screen identifies a vulnerability of neuroendocrine cancer cells to SQLE inhibition
Source: Nat Commun. 2019 Jan 9;10:96. doi: 10.1038/s41467-018-07959-4 (PMC6327044; doi:10.1038/s41467-018-07959-4)
Supplement: Supplementary file 1 — Reporting Summary [file 41467_2018_7959_MOESM1_ESM.pdf]

nature research

Corresponding author(s): Gromoslaw A. Smolen

Reporting Summary

Nature Research wishes to improve the reproducibility of the work that we publish. This form provides structure for consistency and transparency in reporting. For further information on Nature Research policies, see Authors & Referees and the Editorial Policy Checklist.

Statistical parameters

When statistical analyses are reported, confirm that the following items are present in the relevant location (e.g. figure legend, table legend, main text, or Methods section).

n/a	Confirmed	
☐	☒	The exact sample size (*n*) for each experimental group/condition, given as a discrete number and unit of measurement
☐	☒	An indication of whether measurements were taken from distinct samples or whether the same sample was measured repeatedly
☐	☒	The statistical test(s) used AND whether they are one- or two-sided<br>*Only common tests should be described solely by name; describe more complex techniques in the Methods section.*
☒	☐	A description of all covariates tested
☒	☐	A description of any assumptions or corrections, such as tests of normality and adjustment for multiple comparisons
☒	☐	A full description of the statistics including central tendency (e.g. means) or other basic estimates (e.g. regression coefficient) AND variation (e.g. standard deviation) or associated estimates of uncertainty (e.g. confidence intervals)
☐	☒	For null hypothesis testing, the test statistic (e.g. *F*, *t*, *r*) with confidence intervals, effect sizes, degrees of freedom and *P* value noted<br>*Give P values as exact values whenever suitable.*
☒	☐	For Bayesian analysis, information on the choice of priors and Markov chain Monte Carlo settings
☒	☐	For hierarchical and complex designs, identification of the appropriate level for tests and full reporting of outcomes
☒	☐	Estimates of effect sizes (e.g. Cohen's *d*, Pearson's *r*), indicating how they were calculated
☐	☒	Clearly defined error bars<br>*State explicitly what error bars represent (e.g. SD, SE, CI)*

Our web collection on statistics for biologists may be useful.

Software and code

Policy information about availability of computer code

Data collection	Mass spectrometry (metabolite): Peak areas were calculated in El Maven [https://elucidatainc.github.io/ElMaven/] and stable isotopic measurements were corrected against the naturally occurring isotopes for each metabolite measured.<br><br>Mass spectrometry (proteomics): All .RAW files were processed using Proteome Discoverer 2.1.0.81. MS2 spectral assignment was performed using the SEQUEST algorithm using Uniprot Human reference proteome (UP000005640 downloaded 10/05/2016) and a list of known contaminants (CRAPome.org).
Data analysis	Majority of the analyses were performed using GraphPad Prism 7 software package. All data are expressed as mean of multiple measurements (n, indicating the number of replicates). Error bars represent SEM for all in vivo studies and SD for all the in vitro studies. Student’s t test was used to assess statistical significance. Exact values and cutoff are specified within each figure or figure legend.<br><br>Additional statistical analyses and visualizations of cellular growth, RNA-seq, and proteomics data were performed using code written in Python 3.6 using the standard NumPy, pandas, matplotlib, and seaborn packages.

For manuscripts utilizing custom algorithms or software that are central to the research but not yet described in published literature, software must be made available to editors/reviewers upon request. We strongly encourage code deposition in a community repository (e.g. GitHub). See the Nature Research guidelines for submitting code & software for further information.

Data

Policy information about availability of data

All manuscripts must include a data availability statement. This statement should provide the following information, where applicable:

- Accession codes, unique identifiers, or web links for publicly available datasets
- A list of figures that have associated raw data
- A description of any restrictions on data availability

Data supporting the findings of this study are available within this article and supplementary files. The mass spectrometry proteomics data have been deposited in the PRIDE data repository under the accession code PXD011896.

Field-specific reporting

Please select the best fit for your research. If you are not sure, read the appropriate sections before making your selection.

[x] Life sciences [ ] Behavioural & social sciences [ ] Ecological, evolutionary & environmental sciences

For a reference copy of the document with all sections, see nature.com/authors/policies/ReportingSummary-flat.pdf

Life sciences study design

All studies must disclose on these points even when the disclosure is negative.

Sample size	The size of the primary chemical biology screen was determined by the maximum number of available cell lines from a vendor (Zalicus, now part of Horizon Discovery Group).<br>The size of follow up effort in SCLC was determined by the maximum number of available cell lines from common commercial vendors (ATCC, JRCB, DSMZ, Riken BRC).<br>Measurements in miscellaneous in vitro follow up experiments (growth, metabolite accumulation, mRNA expression levels, etc.) were all conducted in triplicates.
Data exclusions	No data were excluded from the analyses.
Replication	The primary screen in 482 cancer cell lines was done once, with each cell line and drug concentration assessed in duplicate. All the subsequent follow up experiments were conducted in triplicates, with several independent biological replications.
Randomization	Randomization is not relevant to our in vitro studies. For in vivo xenograft experiments, mice with tumors within a particular range (150-250 mm3) were randomly assigned to experimental groupings. The goal was to achieve similar mean tumor size at the beginning of the study.
Blinding	Investigators were not blinded to the group allocation during data collection and/or analysis. Blinding was not possible, as the investigators setting up the experiment was also the ones analyzing the data.

Reporting for specific materials, systems and methods

Materials & experimental systems

n/a	Involved in the study	
[x]	[ ]	Unique biological materials
[ ]	[x]	Antibodies
[ ]	[x]	Eukaryotic cell lines
[x]	[ ]	Palaeontology
[ ]	[x]	Animals and other organisms
[x]	[ ]	Human research participants

Methods

n/a	Involved in the study	
[x]	[ ]	ChIP-seq
[x]	[ ]	Flow cytometry
[x]	[ ]	MRI-based neuroimaging

Antibodies

Antibodies used	Primary antibodies used were FDFT1 (13128-1-AP, Proteintech, 1:1000), and β-actin (3700S, Cell Signaling, 1:5000). Secondary antibodies used were IRDye 680RD Donkey anti-Rabbit (926-68073, LI-COR, 1:5000) and IRDye 800CW Donkey anti-Mouse (926-32212, LI-COR, 1:5000).
Validation	Multiple examples of antibody uses are displayed on the vendors' websites. Please refer to specific catalog numbers listed above.

Eukaryotic cell lines

Policy information about cell lines

Cell line source(s)	SCLC cell lines were obtained from the American Type Culture Collection (ATCC), with the following exceptions: LK2 cells were obtained from the Japanese Collection of Research Bioresources Cell Bank (JRCB), LU139 cells from Riken BRC, and SCLC-21H cells from Deutsche Sammlung von Mikroorganismen und Zellkulturen GmbH (DSMZ).
Authentication	Cell lines directly obtained from vendors were not further authenticated.
Mycoplasma contamination	All cell lines were routinely assessed for mycoplasma during the course of the studies and tested negative.
Commonly misidentified lines (See ICLAC register)	None of the commonly misidentified cell lines were used in this study.

Animals and other organisms

Policy information about studies involving animals; ARRIVE guidelines recommended for reporting animal research

Laboratory animals	Five to six week-old female mice were obtained from either Taconic Laboratories (ICR SCID), Jackson Laboratories (NSG) or Charles River Laboratories (Nu/Nu).
Wild animals	The study did not involve wild animals.
Field-collected samples	The study did not involve samples collected from the field.
