# Supplementary material for: A chemical biology screen identifies a vulnerability of neuroendocrine cancer cells to SQLE inhibition
Source: Nat Commun. 2019 Jan 9;10:96. doi: 10.1038/s41467-018-07959-4 (PMC6327044; doi:10.1038/s41467-018-07959-4)
Supplement: Supplementary file 2 — Supplementary Information [file 41467_2018_7959_MOESM2_ESM.pdf]

SUPPLEMENTARY INFORMATION

A chemical biology screen identifies a vulnerability of neuroendocrine cancer cells to SQLE inhibition.

Mahoney, et al.

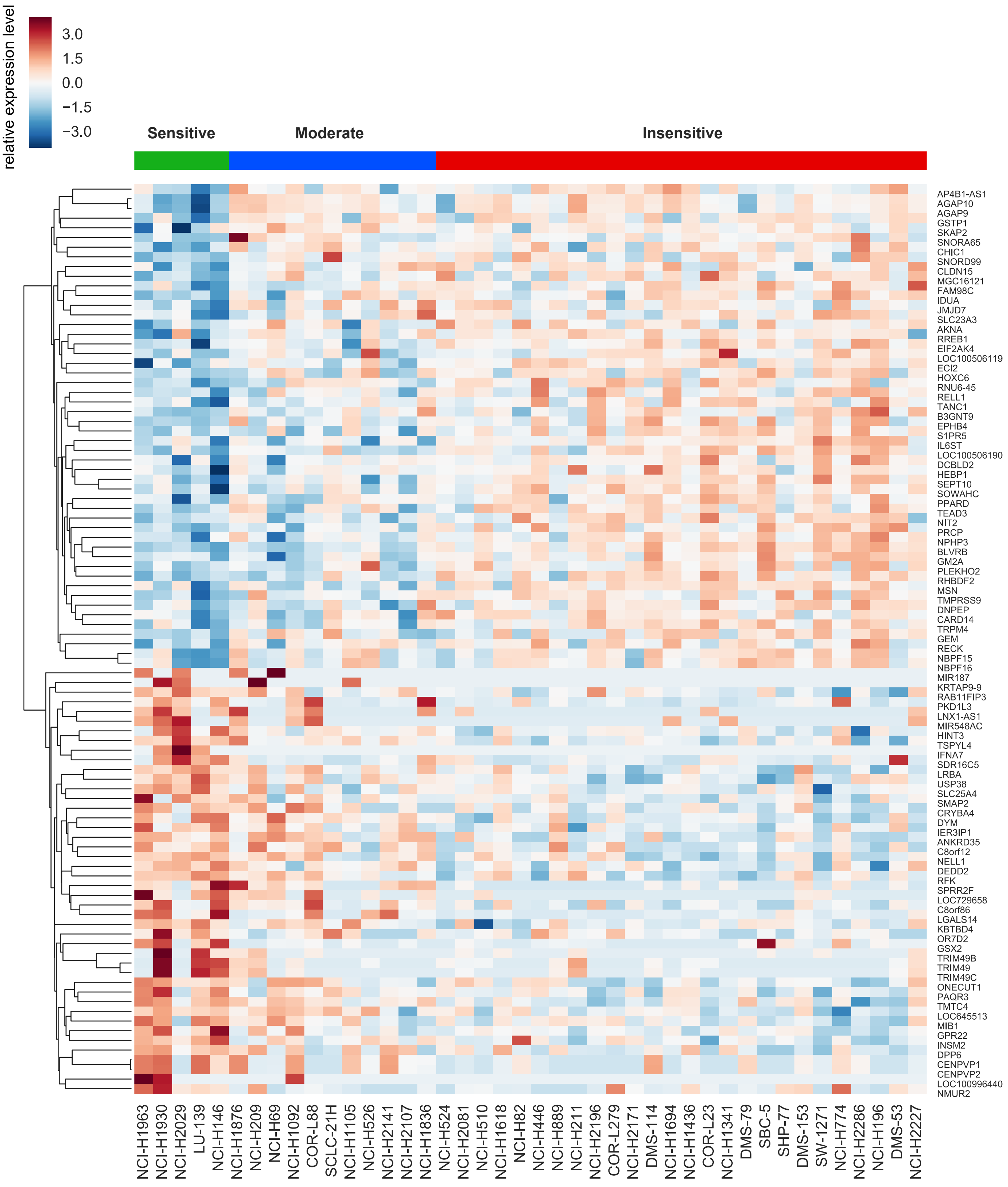

Supplementary Figure 1. RNA-Seq expression signature associated with NB-598 sensitivity developed using 42 SCLC cell lines.

A score for each gene was calculated by unsigned t-test, comparing expression in the sensitive lines against expression in the insensitive cell lines. The top scoring genes (|t|>3.5 for RNA-seq) are displayed in the heatmap. Expression values shown in the heatmap are z-score normalized for each gene.

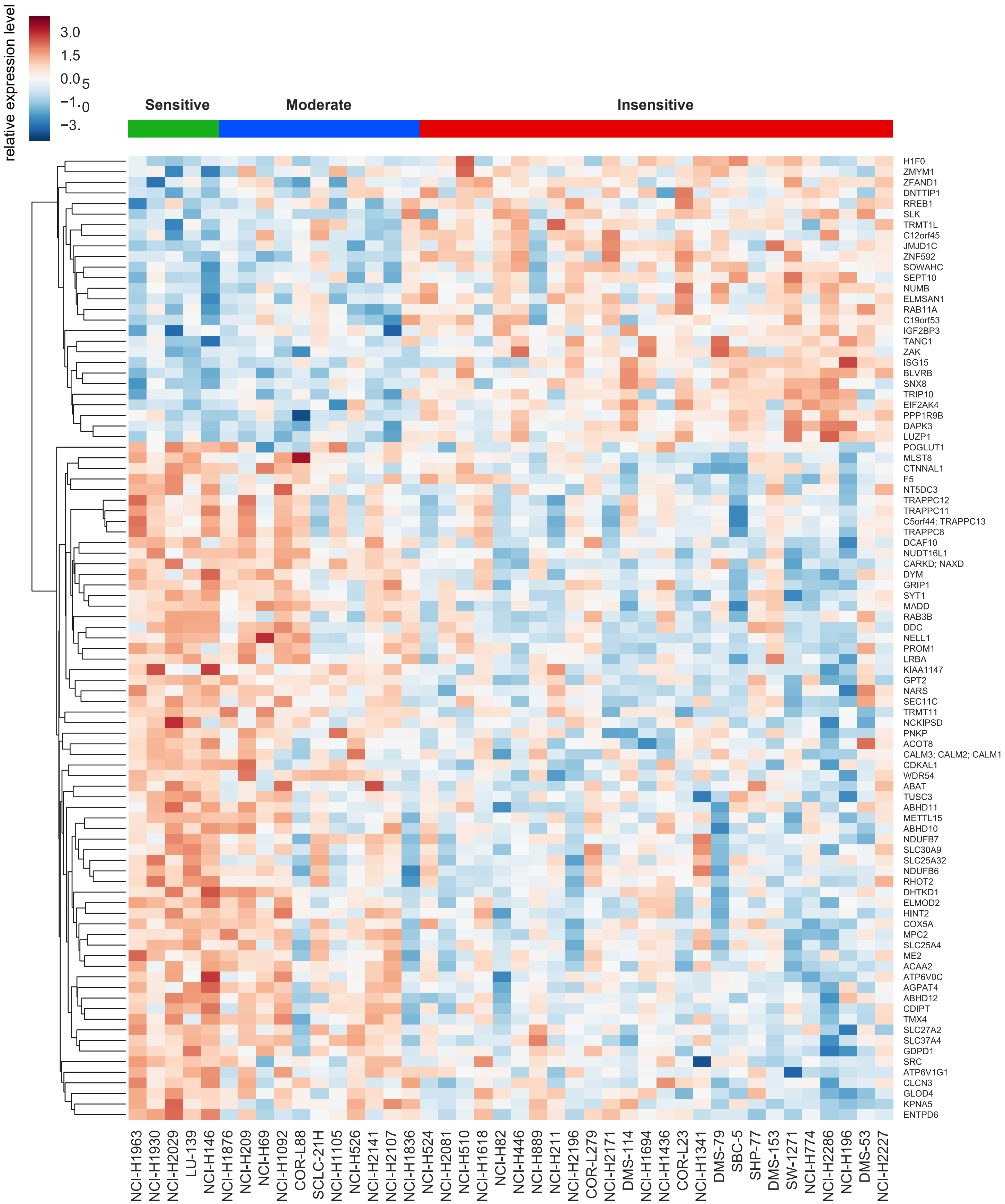

Supplementary Figure 2. Proteomics expression signature associated with NB-598 sensitivity developed using 42 SCLC cell lines.

A score for each gene was calculated by unsigned t-test, comparing expression in the sensitive lines against expression in the insensitive cell lines. The top scoring genes (|t|>3 for proteomics) are displayed in the heatmap. Expression values shown in the heatmap are z-score normalized for each gene.

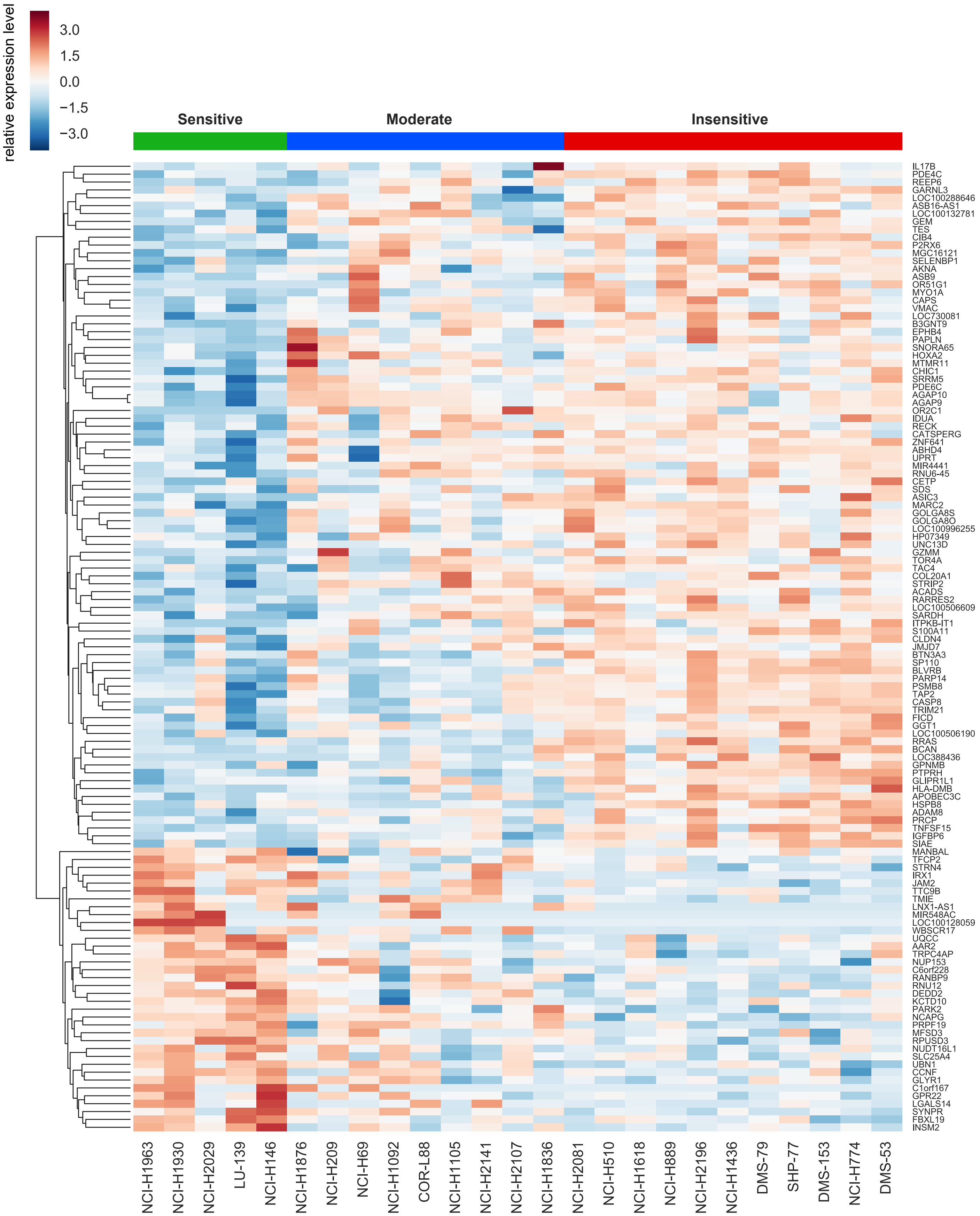

Supplementary Figure 3. RNA-Seq expression signature associated with NB-598 sensitivity developed using 25 cell lines from the ASCL1-high/NEUROD1-low subset.

A score for each gene was calculated by unsigned t-test, comparing expression in the sensitive lines against expression in the insensitive cell lines. The top scoring genes (|t|>3.5 for RNA-seq) are displayed in the heatmap. Expression values shown in the heatmap are z-score normalized for each gene.

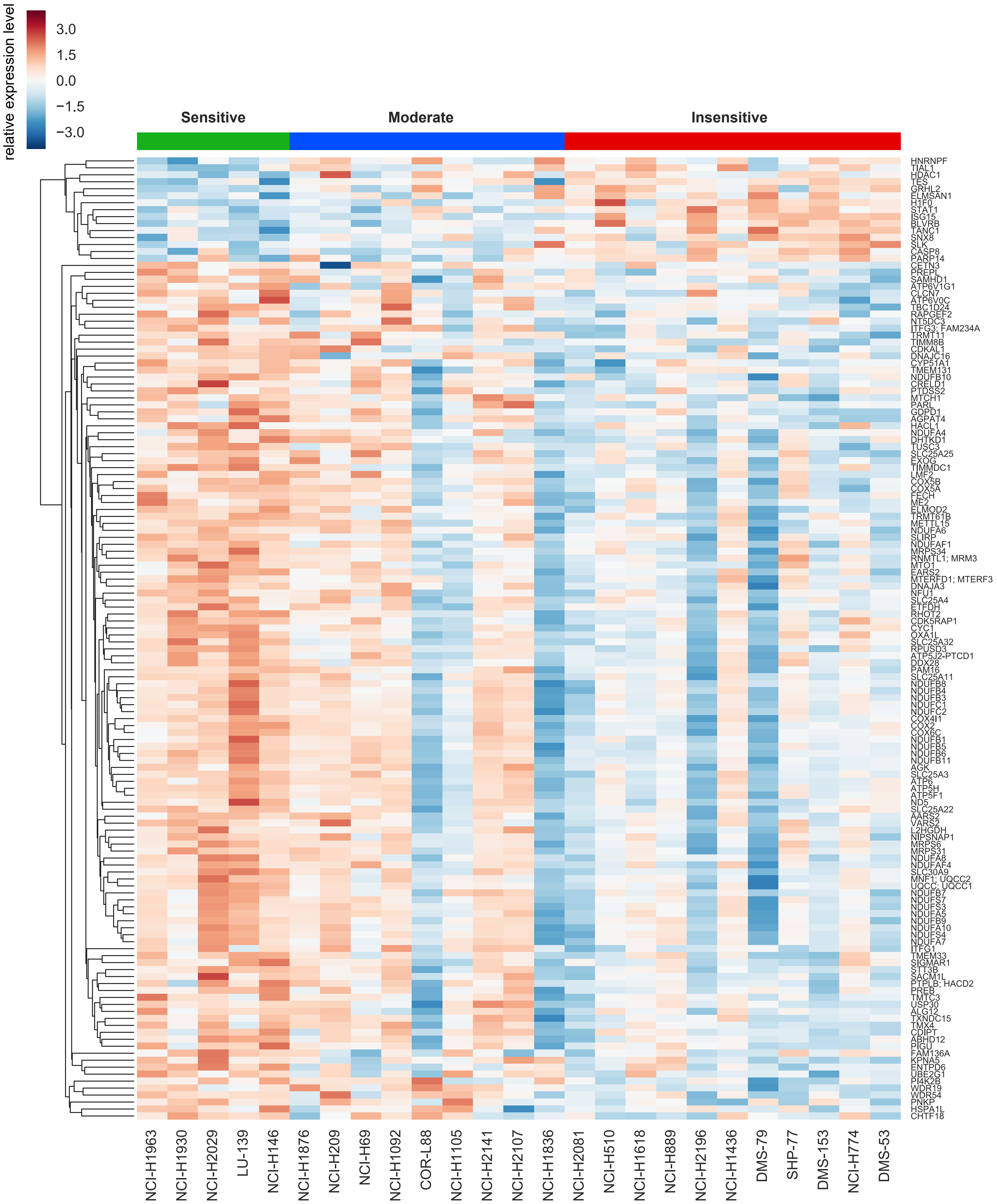

Supplementary Figure 4. Proteomics expression signature associated with NB-598 sensitivity developed using 25 cell lines from the ASCL1-high/NEUROD1-low subset.

A score for each gene was calculated by unsigned t-test, comparing expression in the sensitive lines against expression in the insensitive cell lines. The top scoring genes (|t|>3 for proteomics) are displayed in the heatmap. Expression values shown in the heatmap are z-score normalized for each gene.

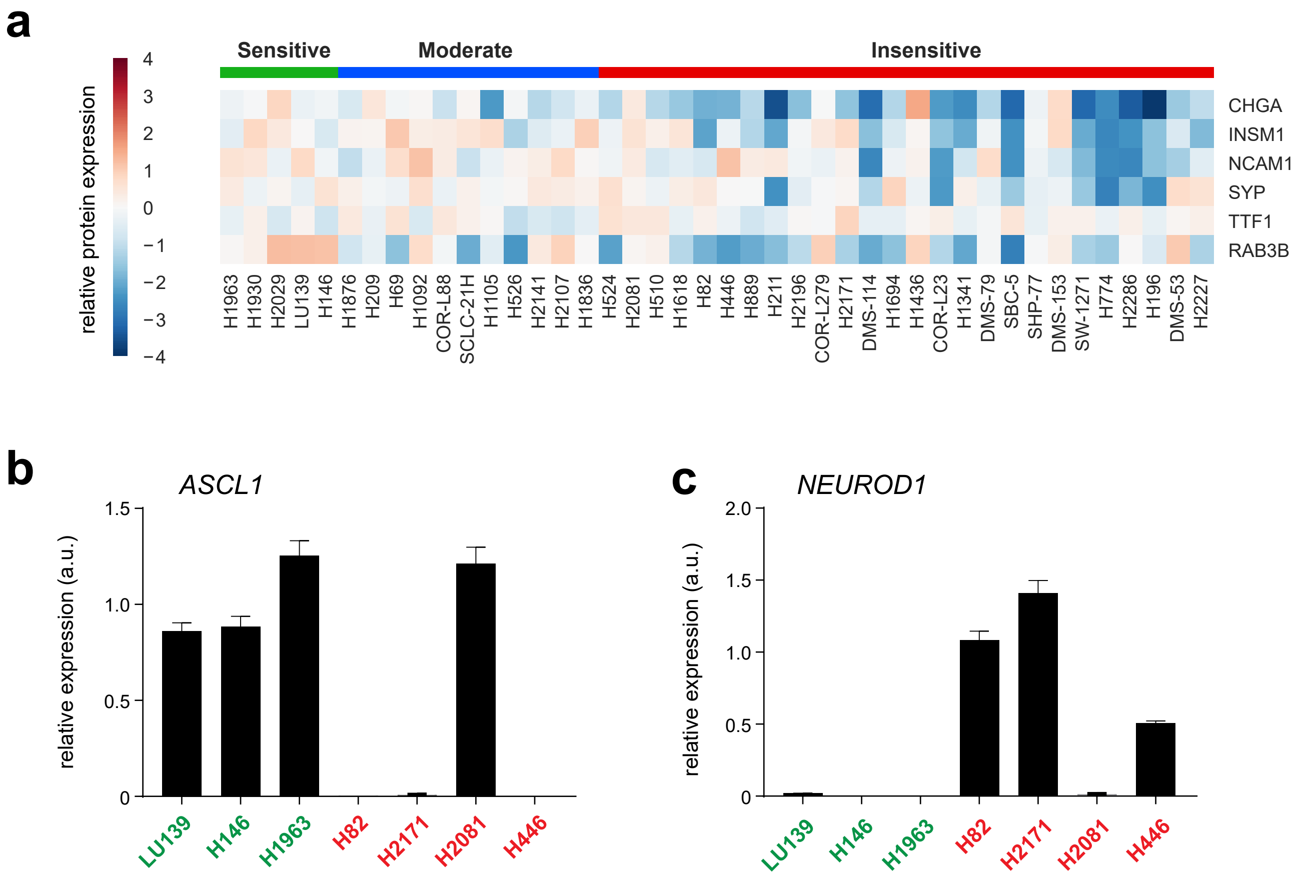

Supplementary Figure 5. Expression of neuroendocrine markers in selected SCLC cell lines.

(**a**) Protein abundance of multiple neuroendocrine markers in SCLC cell lines. The entire proteomics dataset is presented in Supplementary Data 4. Cell lines are color coded based on NB-598 sensitivity, as described in Figure 1, and arranged in order of increasing AUC.

(**b**) Relative *ASCL1* mRNA expression levels in a representative panel of SCLC cell lines. Mean values of triplicate measurements from a representative experiment are plotted and error bars represent s.d..

(**c**) Relative *NEUROD1* mRNA expression levels in a representative panel of SCLC cell lines. Mean values of triplicate measurements from a representative experiment are plotted and error bars represent s.d..

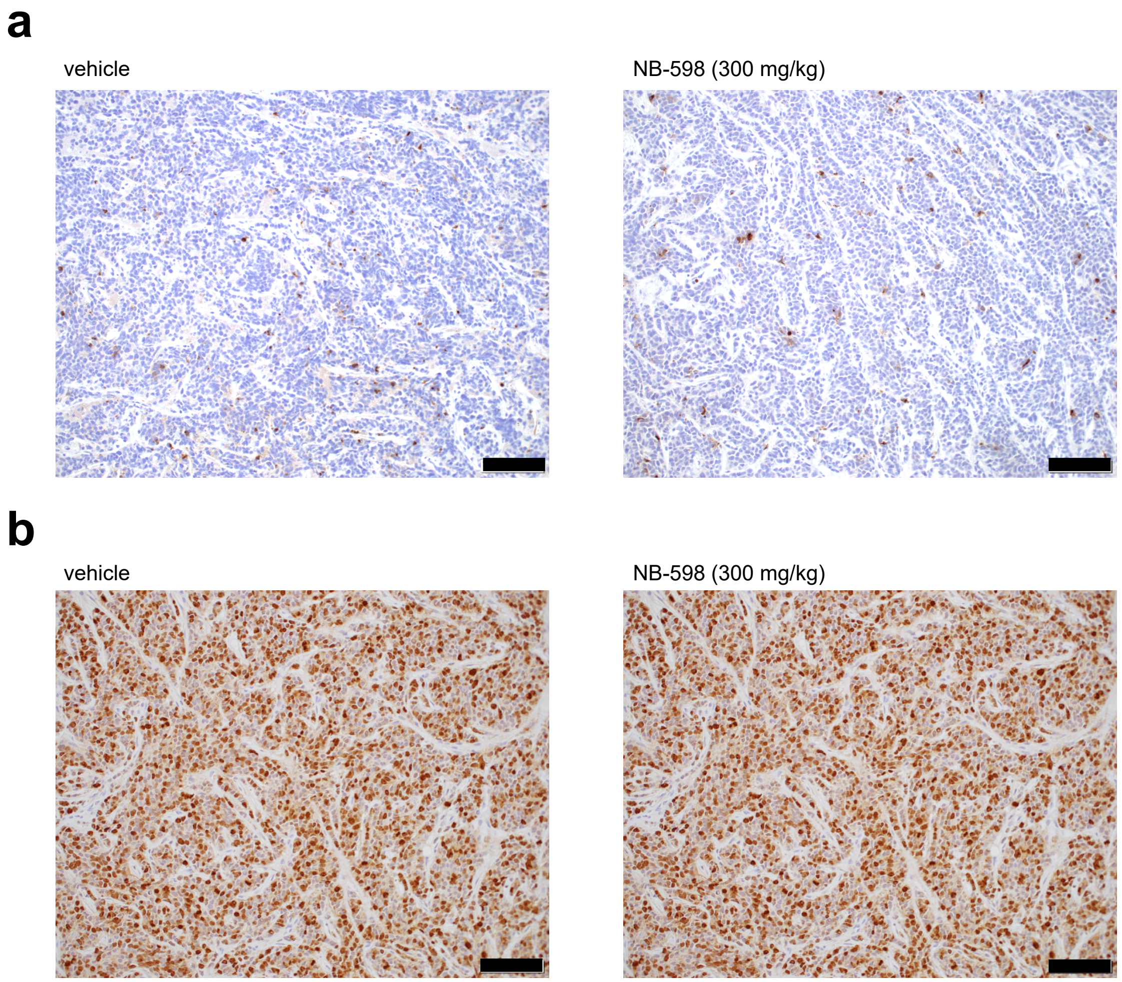

Supplementary Figure 6. Immunohistological assessment of H1963 tumors at the end of the efficacy study.

(**a**) Representative image of tumor sections stained for cleaved caspase 3, an apoptosis marker. Scale bar corresponds to 100μm.

(**b**) Representative image of tumor sections stained for Ki-67, a proliferation marker. Scale bar corresponds to 100μm.

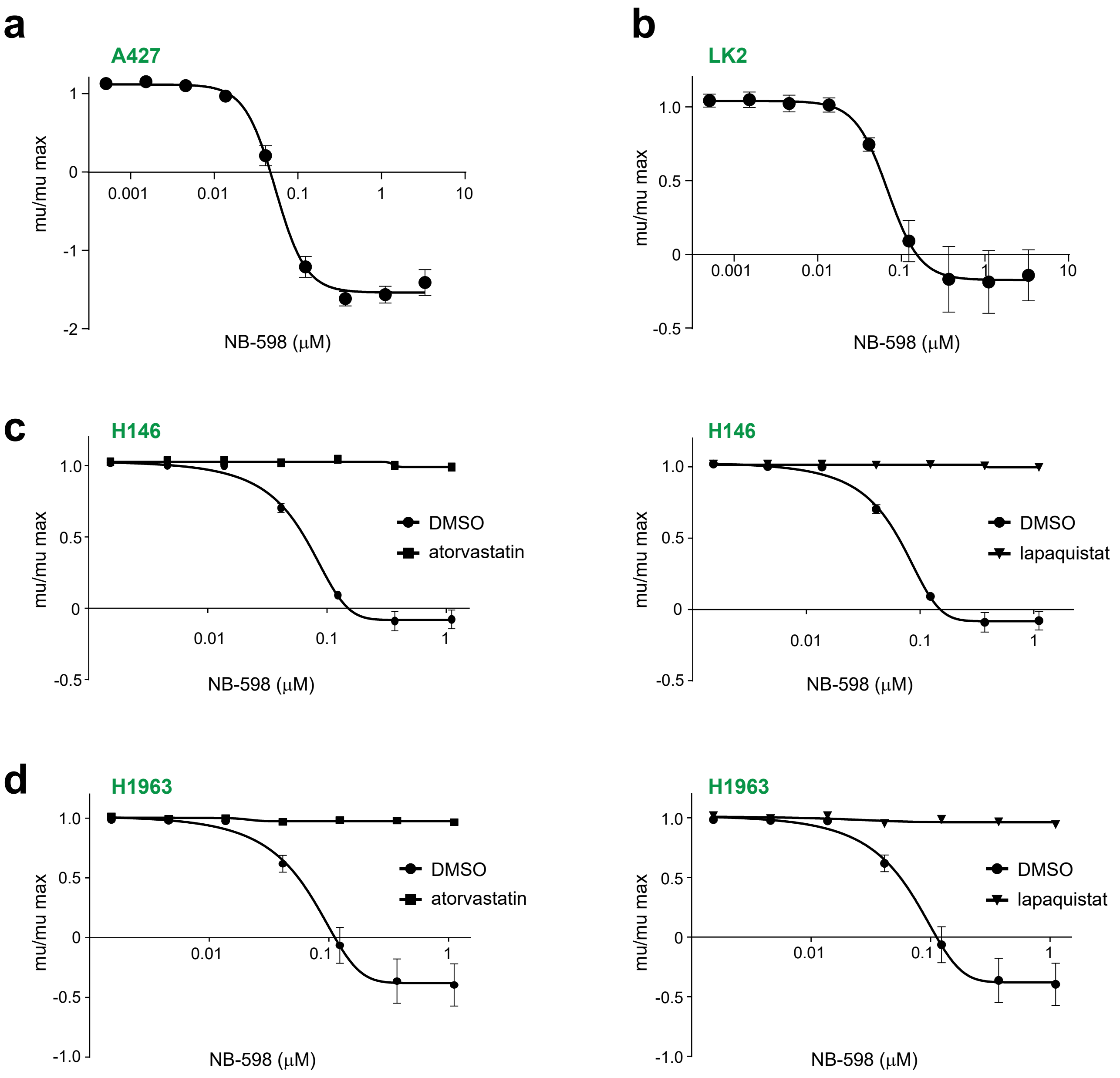

Supplementary Figure 7. Additional data supporting Figure 5.

(**a**) NB-598 sensitivity of the A427 cell line.

(**b**) NB-598 sensitivity of the LK2 cell line.

(**c**) Pharmacological suppression of NB-598 growth defects in H146 cells using 10μM atorvastatin or 10μM lapaquistat.

(**d**) Pharmacological suppression of NB-598 growth defects in H1963 cells using 10μM atorvastatin or 10μM lapaquistat.

For all panels, mean values of triplicate measurements from a representative experiment are plotted and error bars represent s.d..

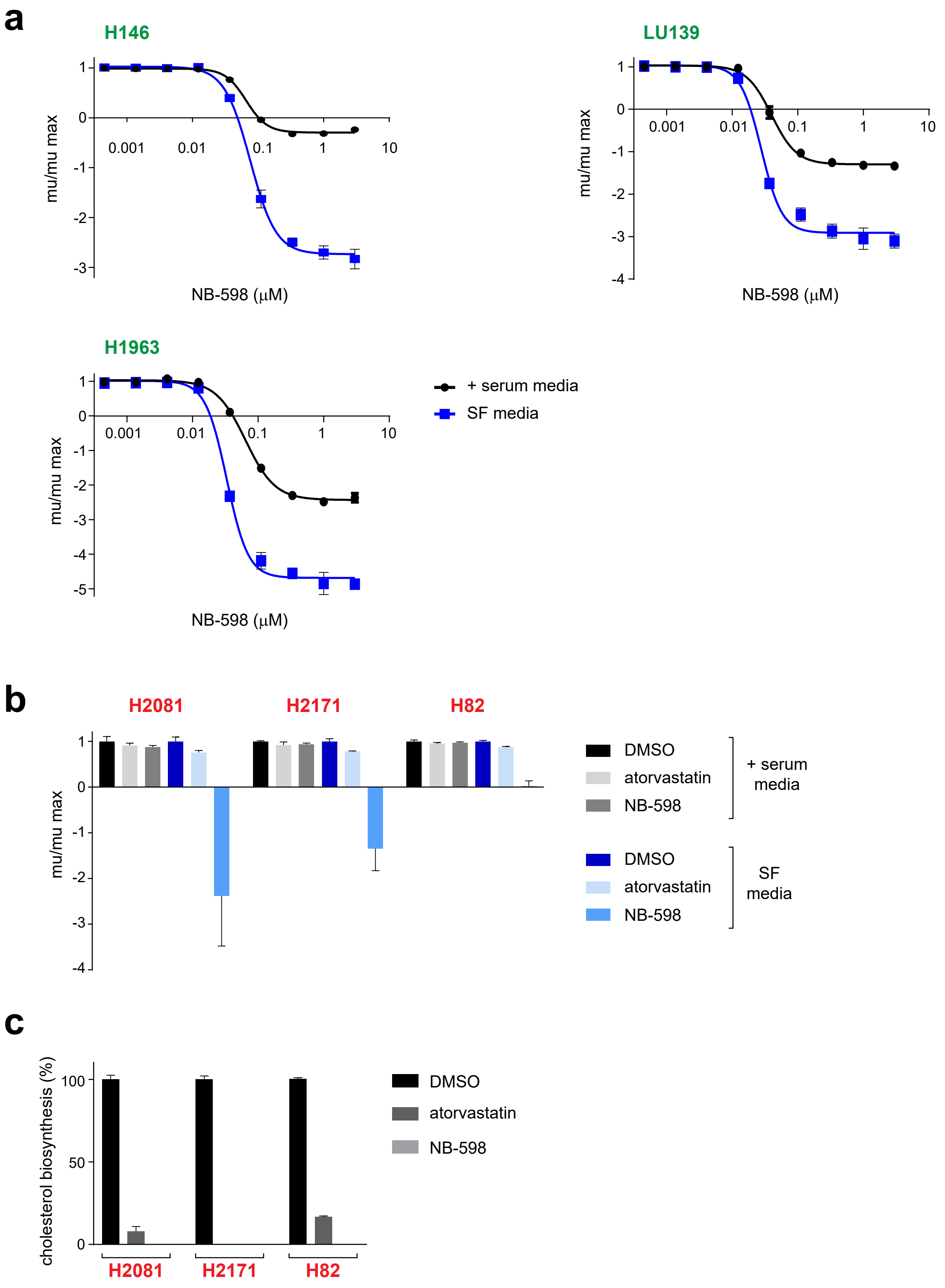

Supplementary Figure 8. Additional data supporting Figure 6.

(**a**) Relative NB-598 sensitivity in a representative panel of SQLE-sensitive cell lines grown in two different media: serum-containing and serum-free (SF). Time of treatment was 72h.

(**b**) Relative sensitivity to NB-598 and atorvastatin of SQLE-insensitive cell lines grown in two different media: serum-containing and serum-free (SF). Time of treatment was 72h.

(**c**) Relative *de novo* cholesterol biosynthesis inhibition measured in multiple SQLE-insensitive cell lines using $^{13}C_2$-acetate labeling. Effects on cholesterol m+2 isotopomer were quantitated.

For all panels, mean values of triplicate measurements from a representative experiment are plotted and error bars represent s.d..

a

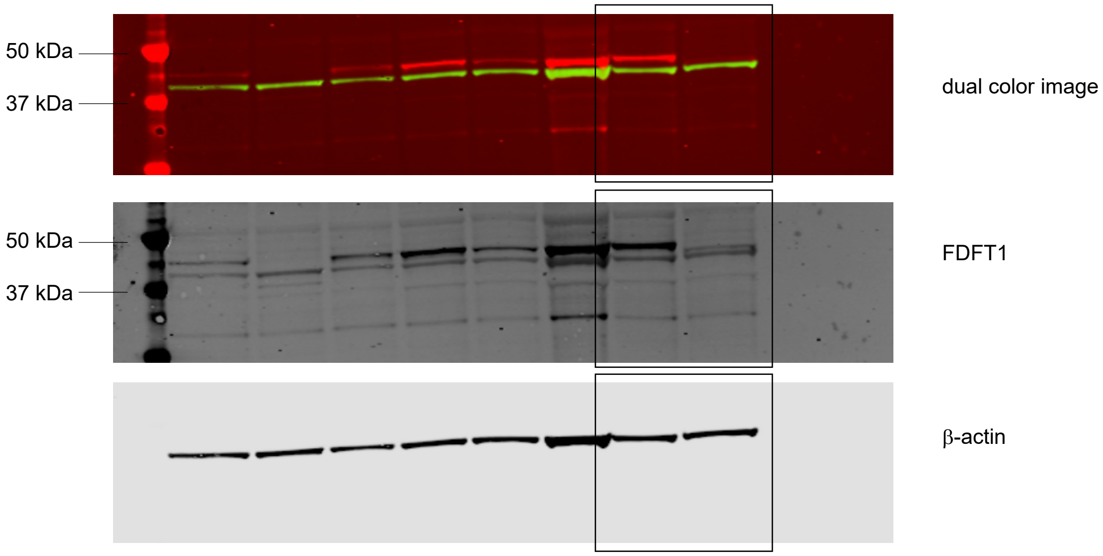

b

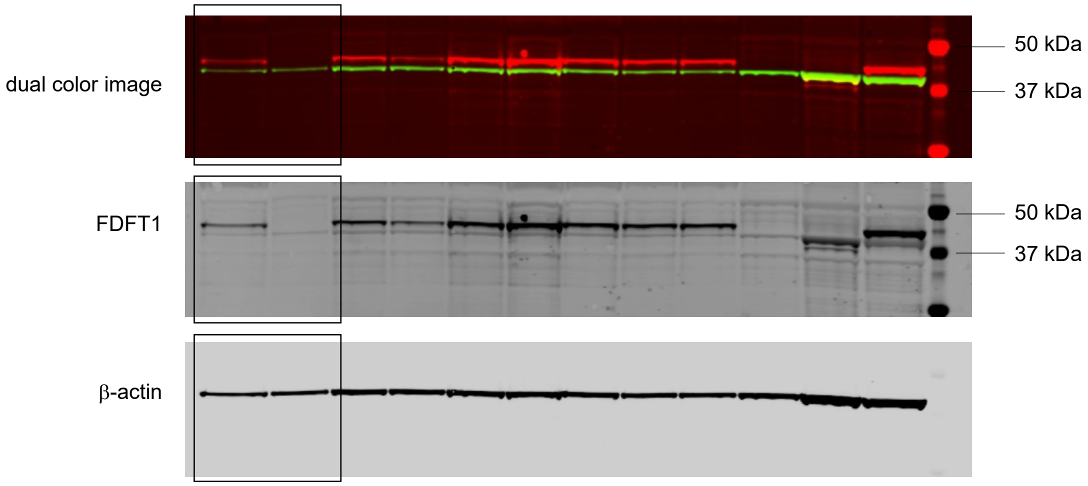

Supplementary Figure 9. Uncropped images of immunoblots displayed in Figure 5b.

(**a**) Ucropped images corresponding to immunoblot analyses of A427 cells.

(**b**) Ucropped images corresponding to immunoblot analyses of LK2 cells.
