# Supplementary material for: A chemical biology screen identifies a vulnerability of neuroendocrine cancer cells to SQLE inhibition
Source: Nat Commun. 2019 Jan 9;10:96. doi: 10.1038/s41467-018-07959-4 (PMC6327044; doi:10.1038/s41467-018-07959-4)
Supplement: Supplementary file 3 — Description of Additional Supplementary Files [file 41467_2018_7959_MOESM3_ESM.docx]

**Title:** Supplementary Data 1
**Description:** NB-598 sensitivity in a panel of 482 cell lines.

**Title:** Supplementary Data 2
**Description:** NB-598 sensitivity in a panel of 42 SCLC cell lines.

**Title:**  Supplementary Data 3
**Description:** RNA-seq dataset for 42 SCLC cell lines. Values represent log2-transformed normalized counts.

**Title:** Supplementary Data 4
**Description:**  Proteomics dataset for 42 SCLC cell lines. Values represent the natural log of the ratio between the specified cell line sample and a pooled sample from all 42 lines. Values provided are the average of typically 3 biological replicates.

**Title:**  Supplementary Data 5
**Description:** CRISPR suppressor screen results.
